# Supplementary figures and images for: Epstein-Barr virus reactivation induces divergent abortive, reprogrammed, and host shutoff states by lytic progression
Source: PLoS Pathog. 2024 Oct 24;20(10):e1012341. doi: 10.1371/journal.ppat.1012341 (PMC11563402; doi:10.1371/journal.ppat.1012341)

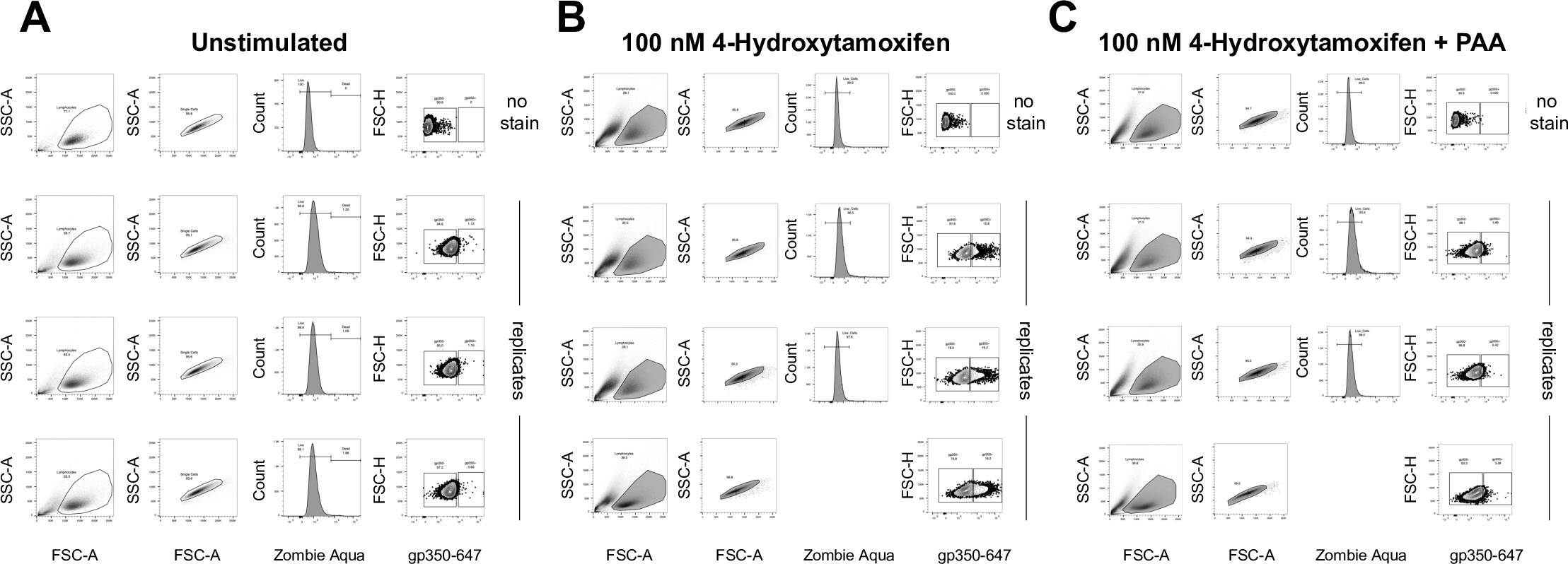

Supplement: S1 Fig — (A) Lymphocyte, singlet, live-cell, and gp350+ gating for unstimulated cells. (B) The same gating strategy as above applied for 4HT-treated cells. (C) The same gating strategy as above applied for cells co-treated with 4HT and PAA. (TIF) [file ppat.1012341.s001.tif]

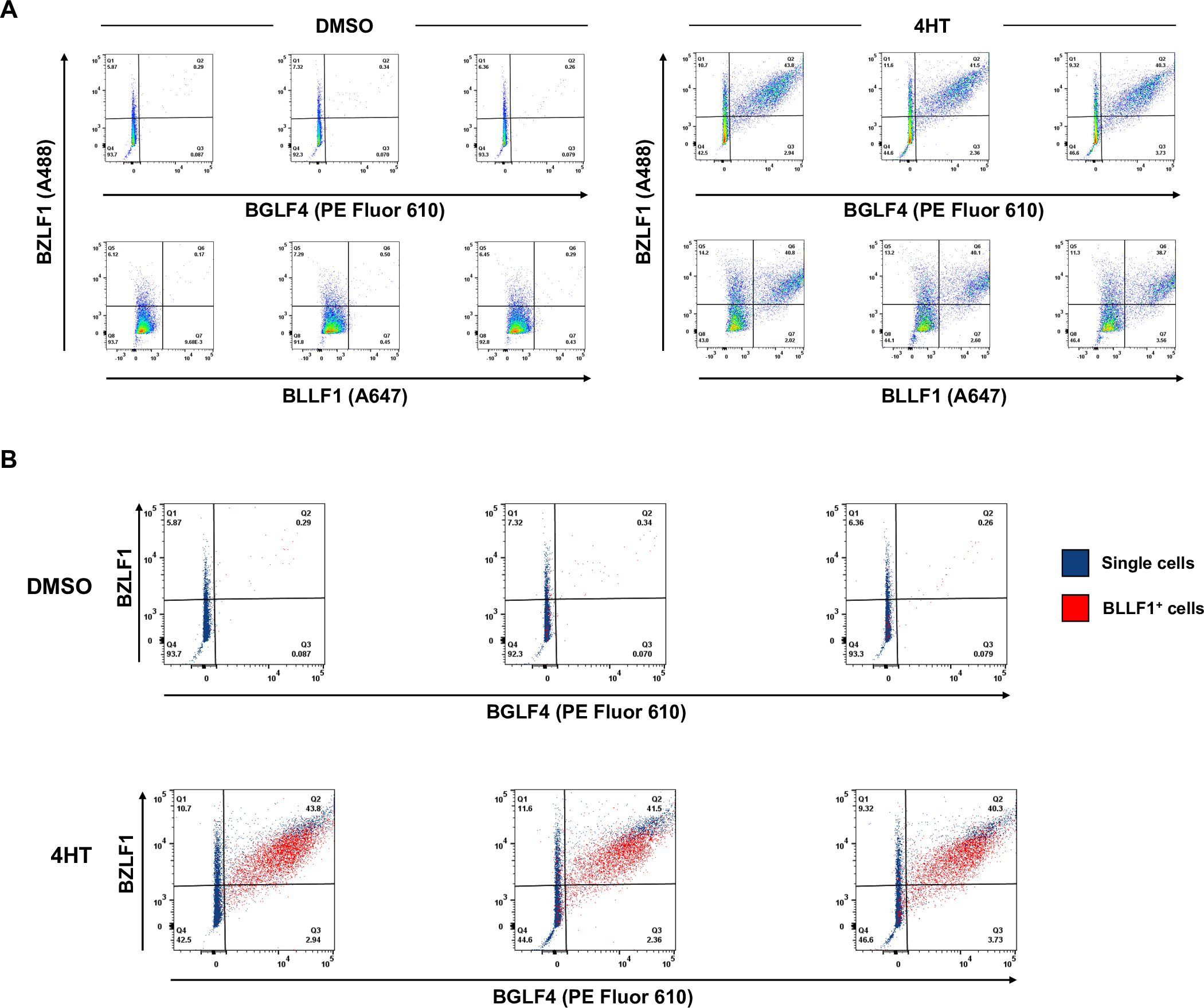

Supplement: S2 Fig — (A) Co-expression of BZLF1 with BGLF4 or BLLF1 in DMSO control treatment and 4HT-induced reactivation. (B) Co-expression of BZLF1, BGLF4, and BLLF1 (red cells) in DMSO control treatment and 4HT-induced reactivation. (TIF) [file ppat.1012341.s002.tif]

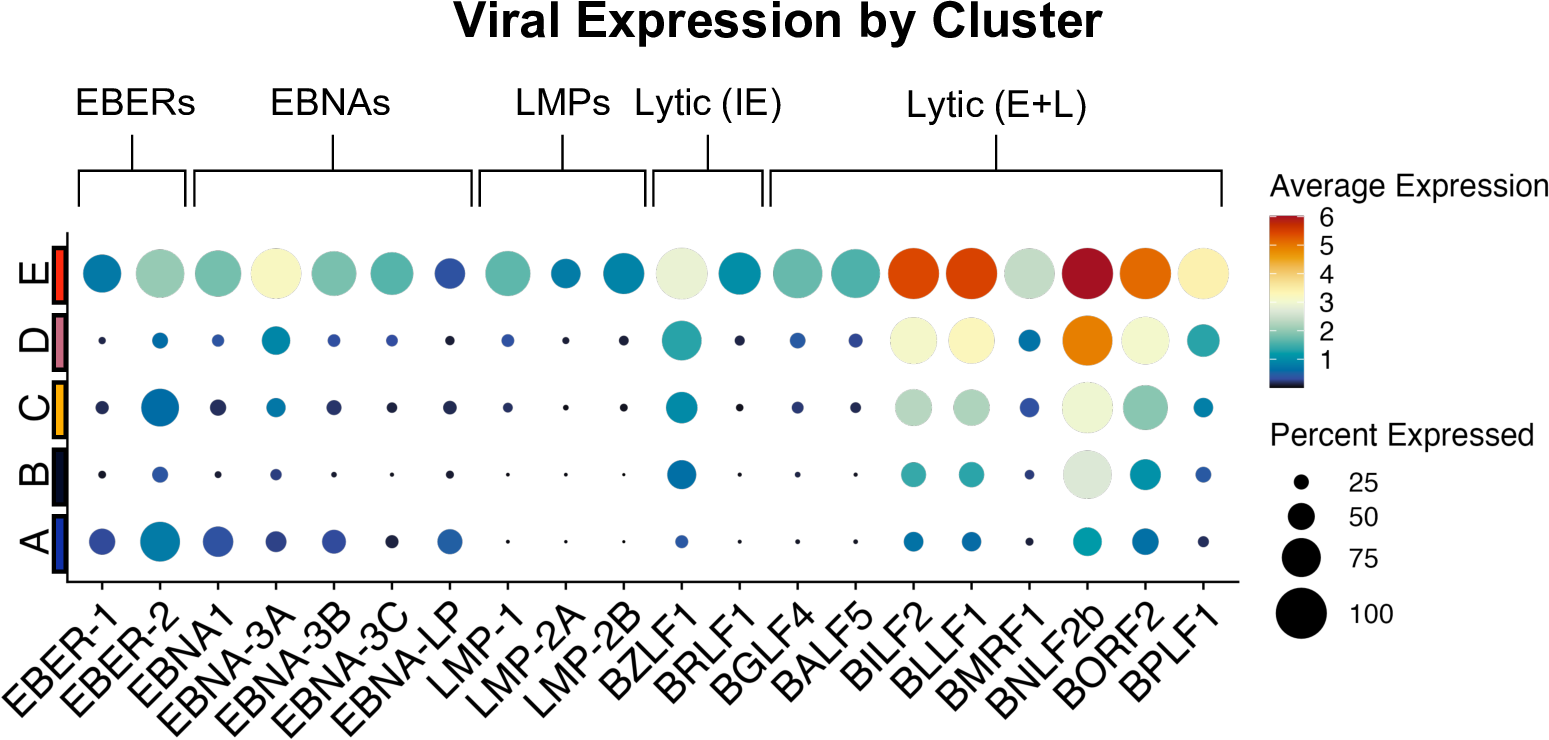

Supplement: S3 Fig — (TIF) [file ppat.1012341.s003.tif]

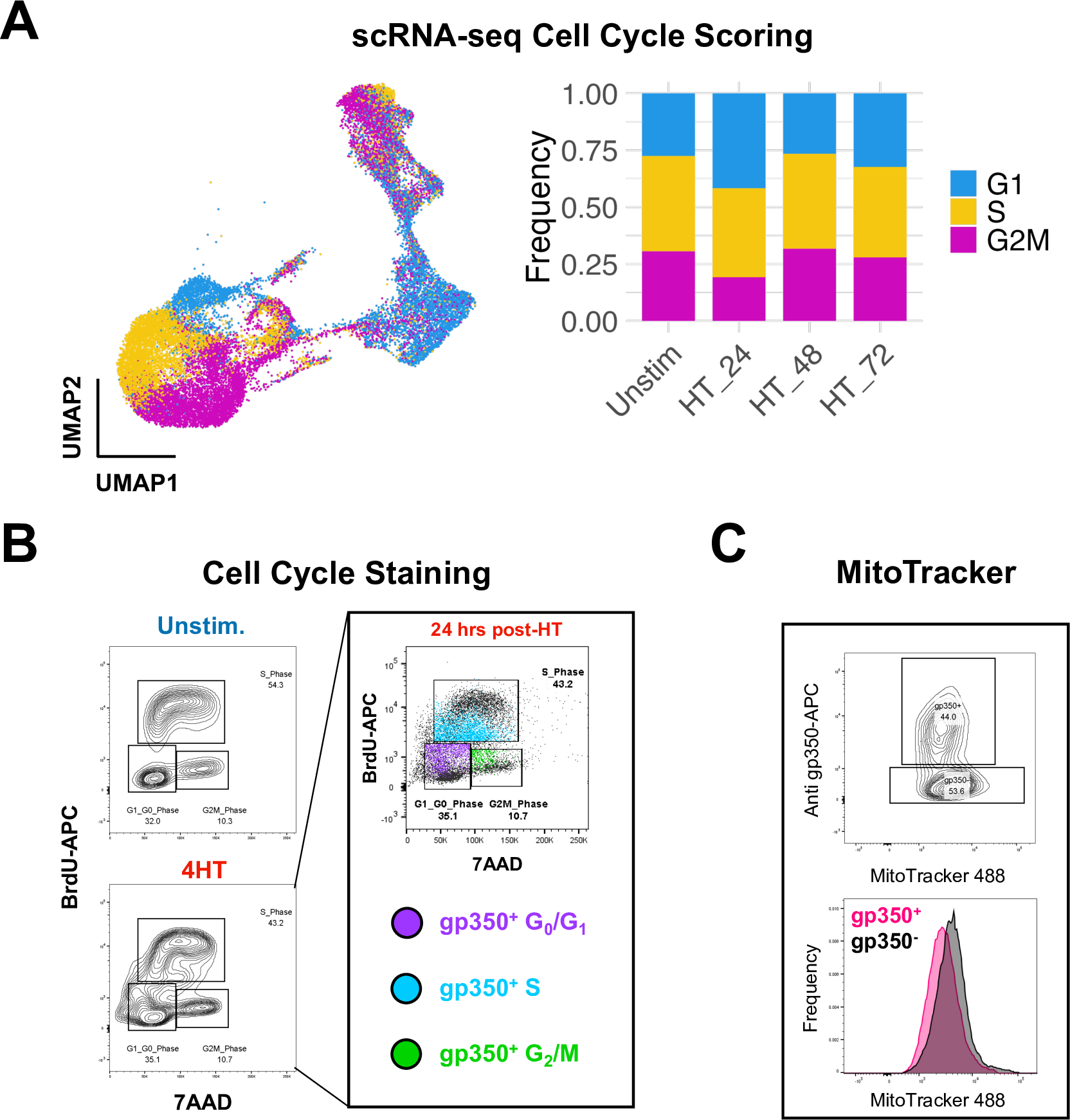

Supplement: S4 Fig — (A) Cell cycle phase annotations in P3HR1-ZHT scRNA-seq data. (B) Flow cytometry cell cycle analysis in unstimulated and 4HT-treated P3HR1-ZHT cells with gp350+ cells highlighted. (C) MitoTracker staining by gp350 status in 4HT-treated P3HR1-ZHT cells. (TIF) [file ppat.1012341.s004.tif]

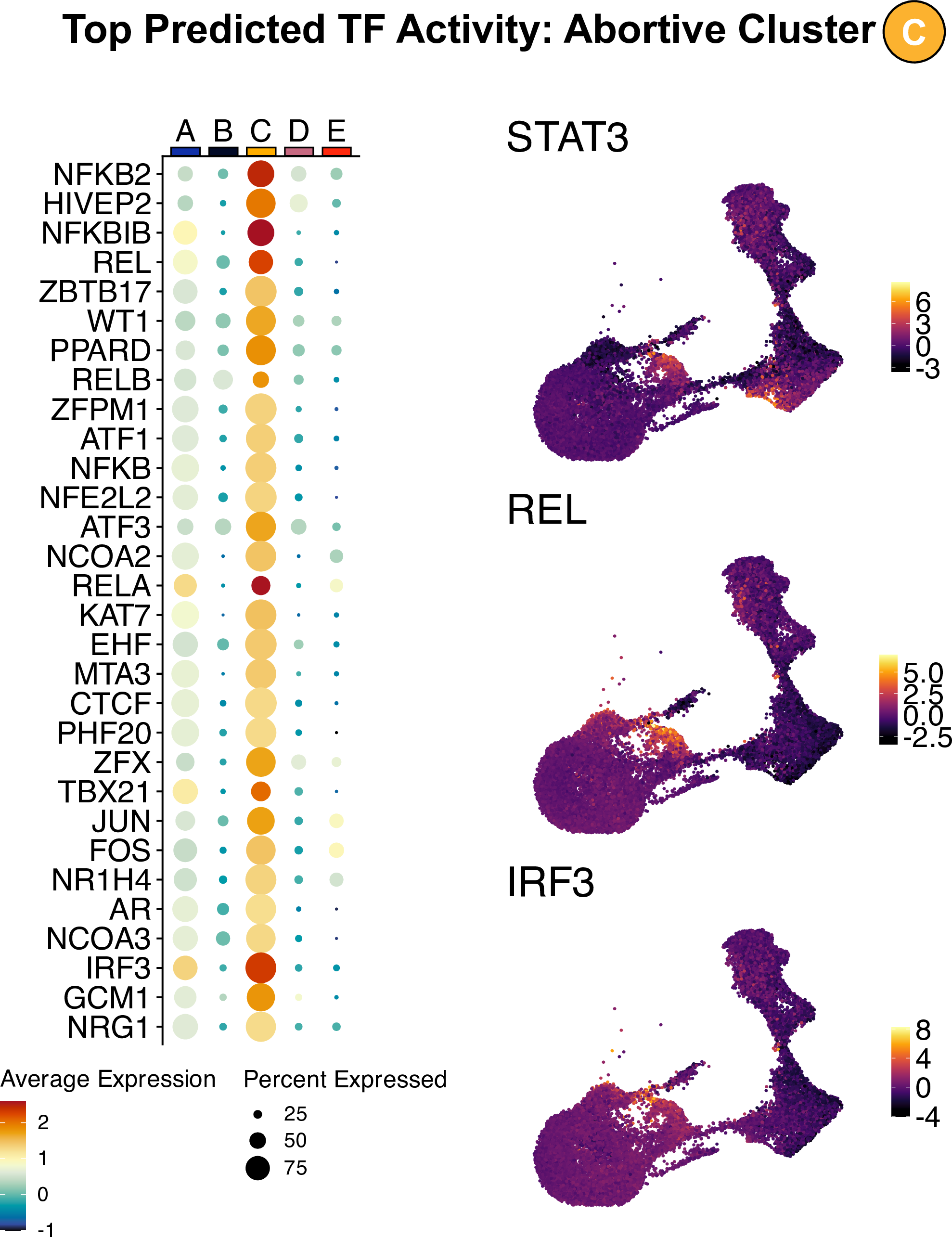

Supplement: S5 Fig — (TIF) [file ppat.1012341.s005.tif]

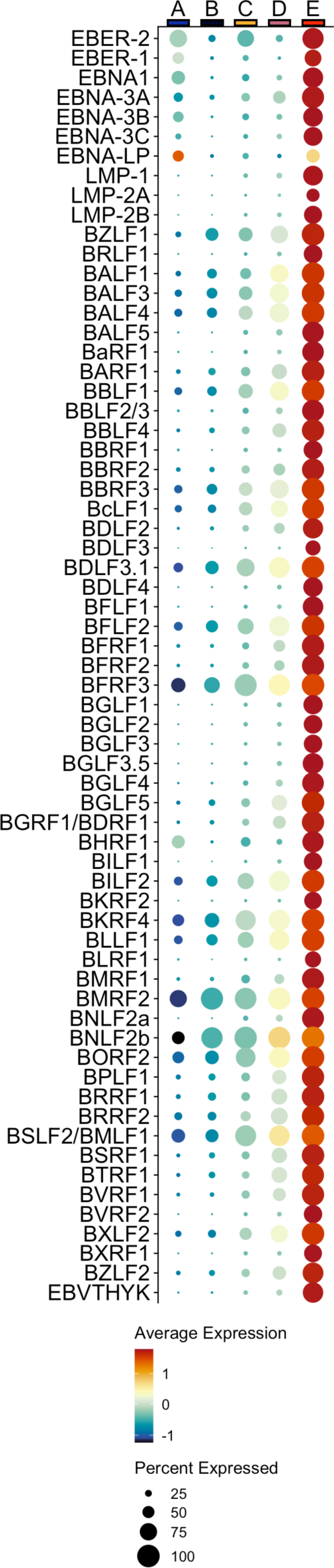

Supplement: S6 Fig — (TIF) [file ppat.1012341.s006.tif]

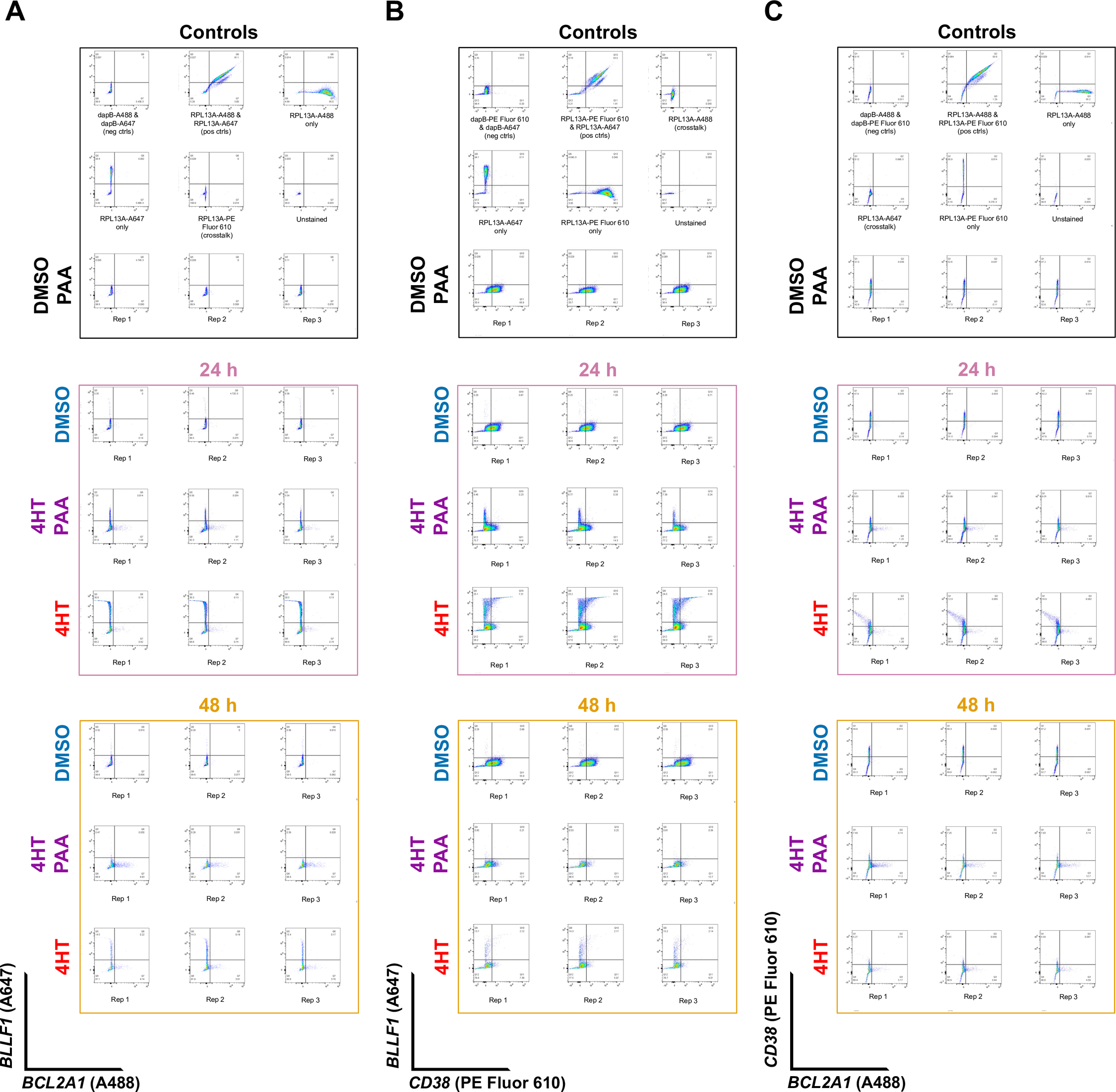

Supplement: S7 Fig — (A) Technical controls, 24 h, and 48 h responses to DMSO, 4HT, and 4HT+PAA for BCL2A1 versus BLLF1 expression. (B) Technical controls, 24 h, and 48 h responses to DMSO, 4HT, and 4HT+PAA for CD38 versus BLLF1 expression. (C) Technical controls, 24 h, and 48 h responses to DMSO, 4HT, and 4HT+PAA for BCL2A1 versus CD38 expression. (TIF) [file ppat.1012341.s007.tif]

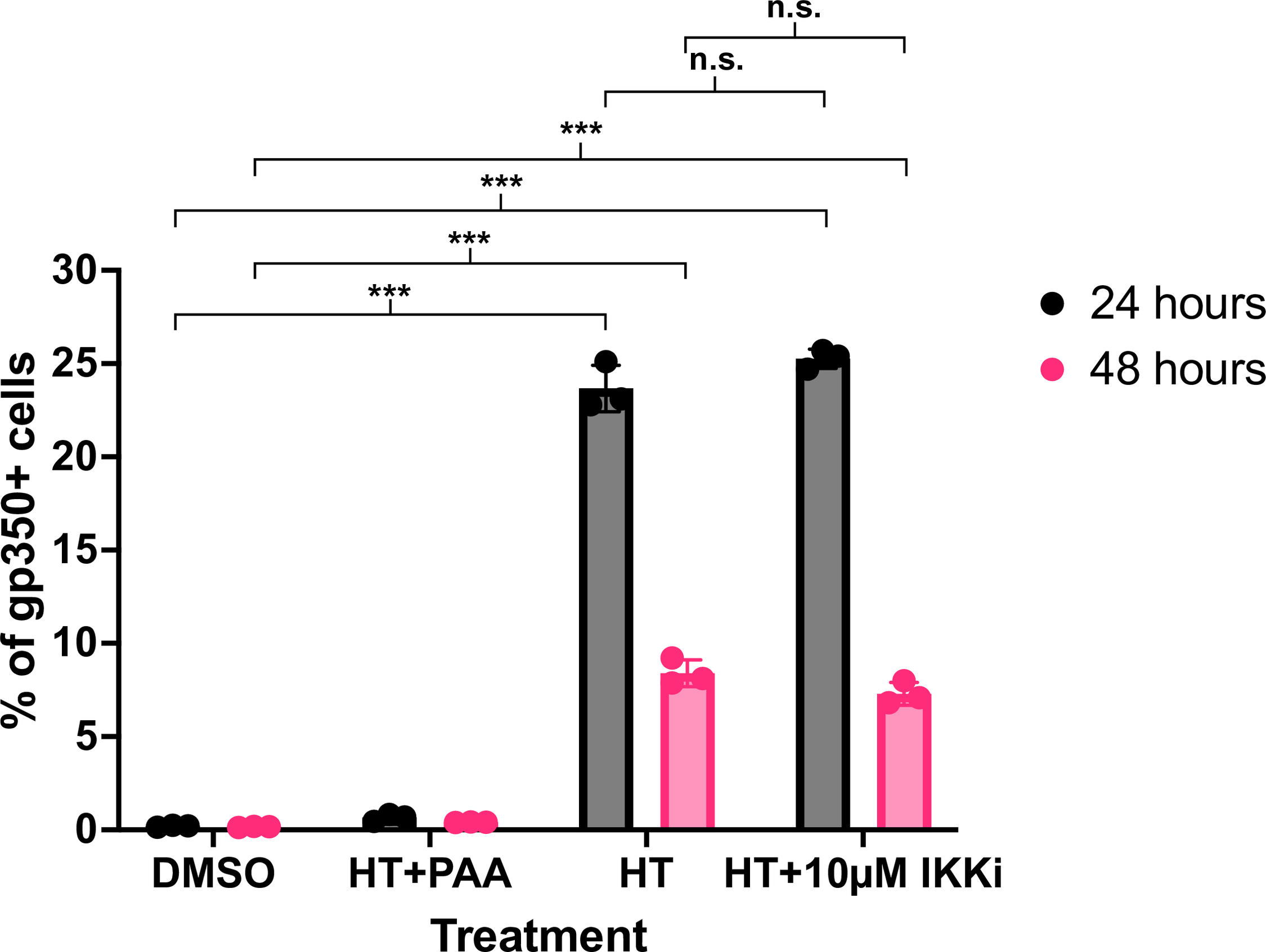

Supplement: S8 Fig — Statistical comparisons between groups (n = 3 replicates per treatment condition) were evaluated via Welch’s two-tailed t tests (***p<0.001) (TIF) [file ppat.1012341.s008.tif]

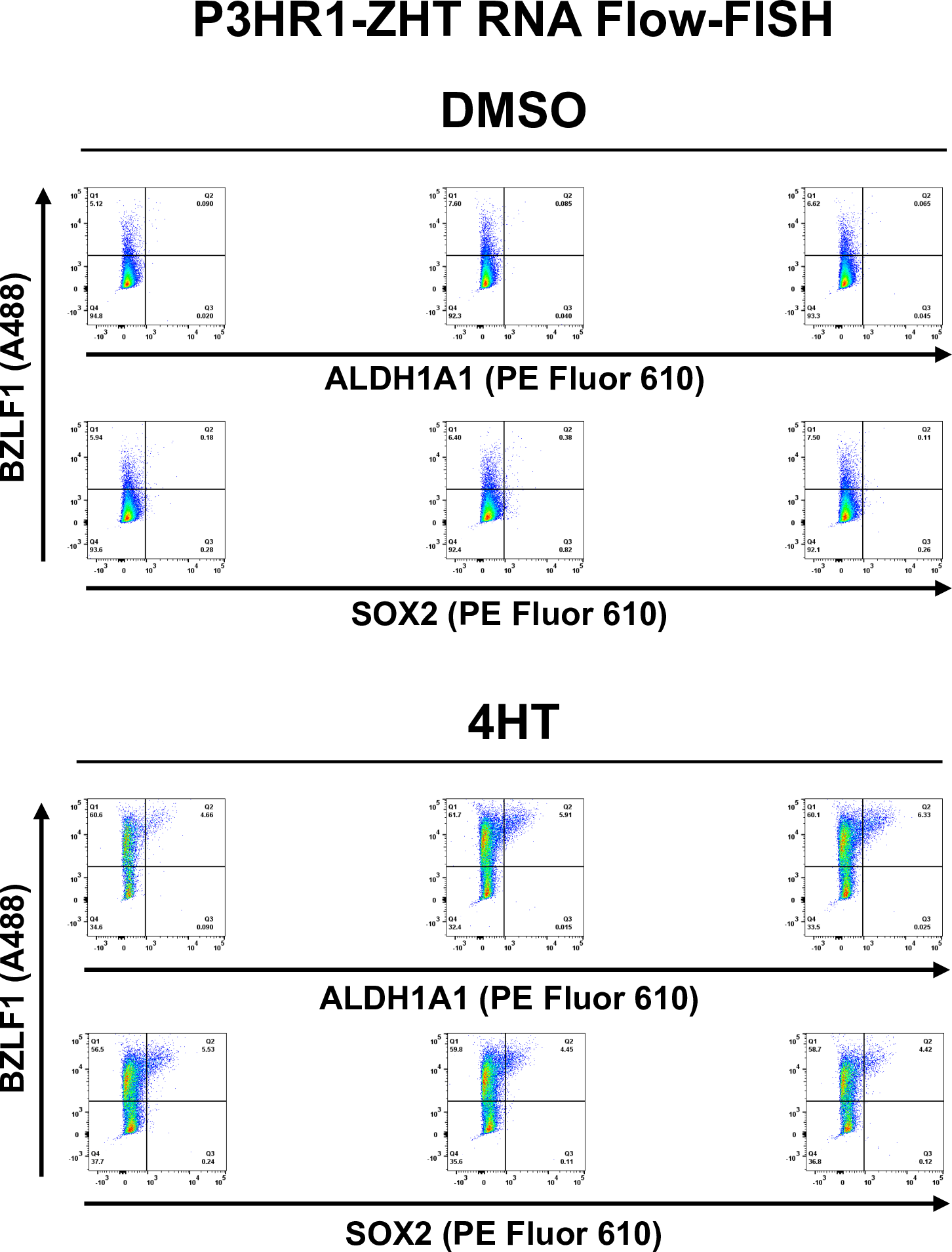

Supplement: S9 Fig RNA — (TIF) [file ppat.1012341.s009.tif]

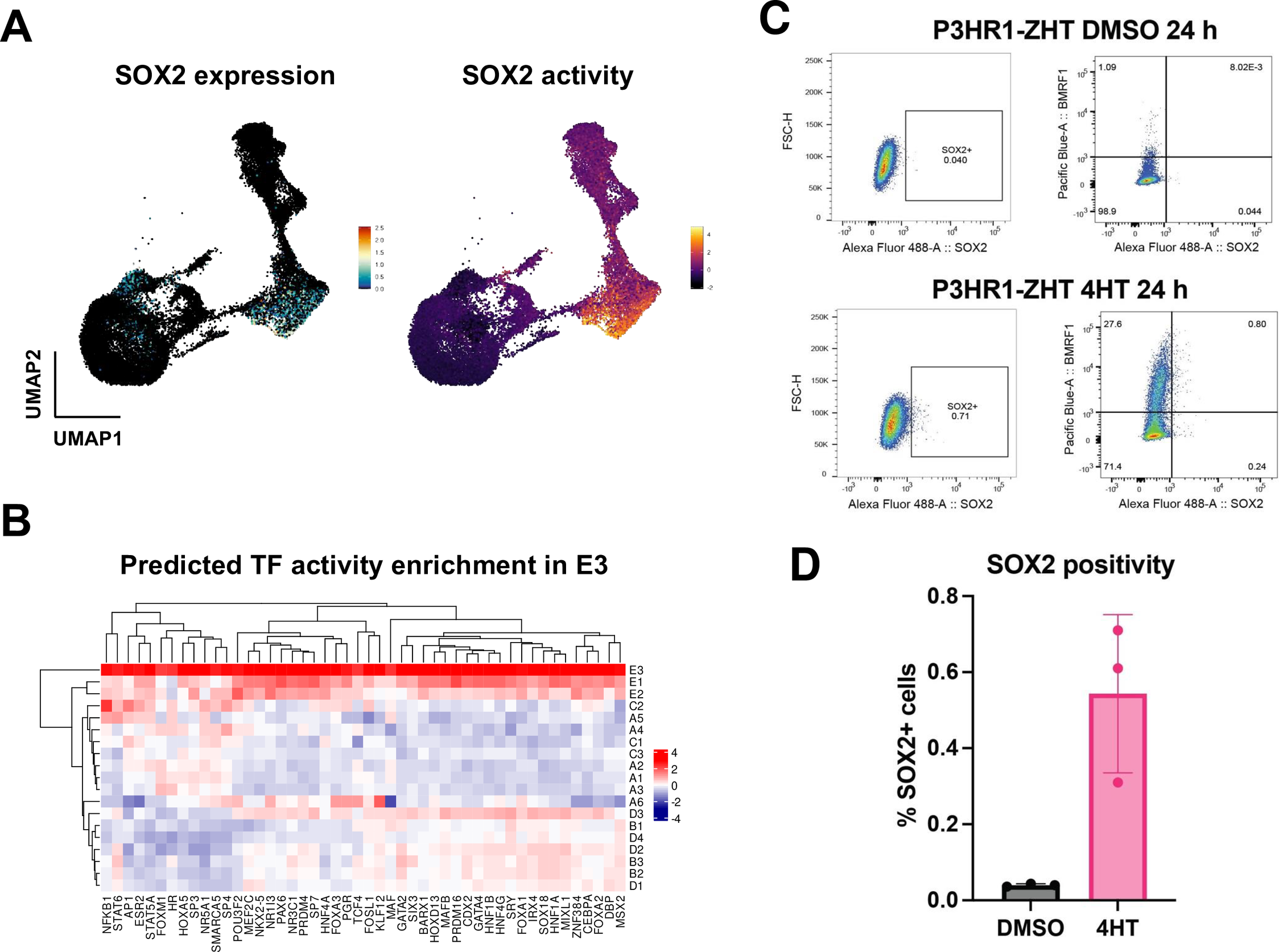

Supplement: S10 Fig — (A) SOX2 scRNA-seq expression and gene regulatory network activity. (B) Hierarchical clustering of predicted TF activities by P3HR1-ZHT subcluster. (C) Flow plots of untreated and treated P3HR1-ZHT cells 24 h post stimulation. Flow plots show that a small percentage of induced cells express SOX2 and that most of these cells are lytic (BMRF1+). (D) Bar graph depicting the percentage of cells that expressed SOX2 between treatment groups, in biological triplicate. (TIF) [file ppat.1012341.s010.tif]

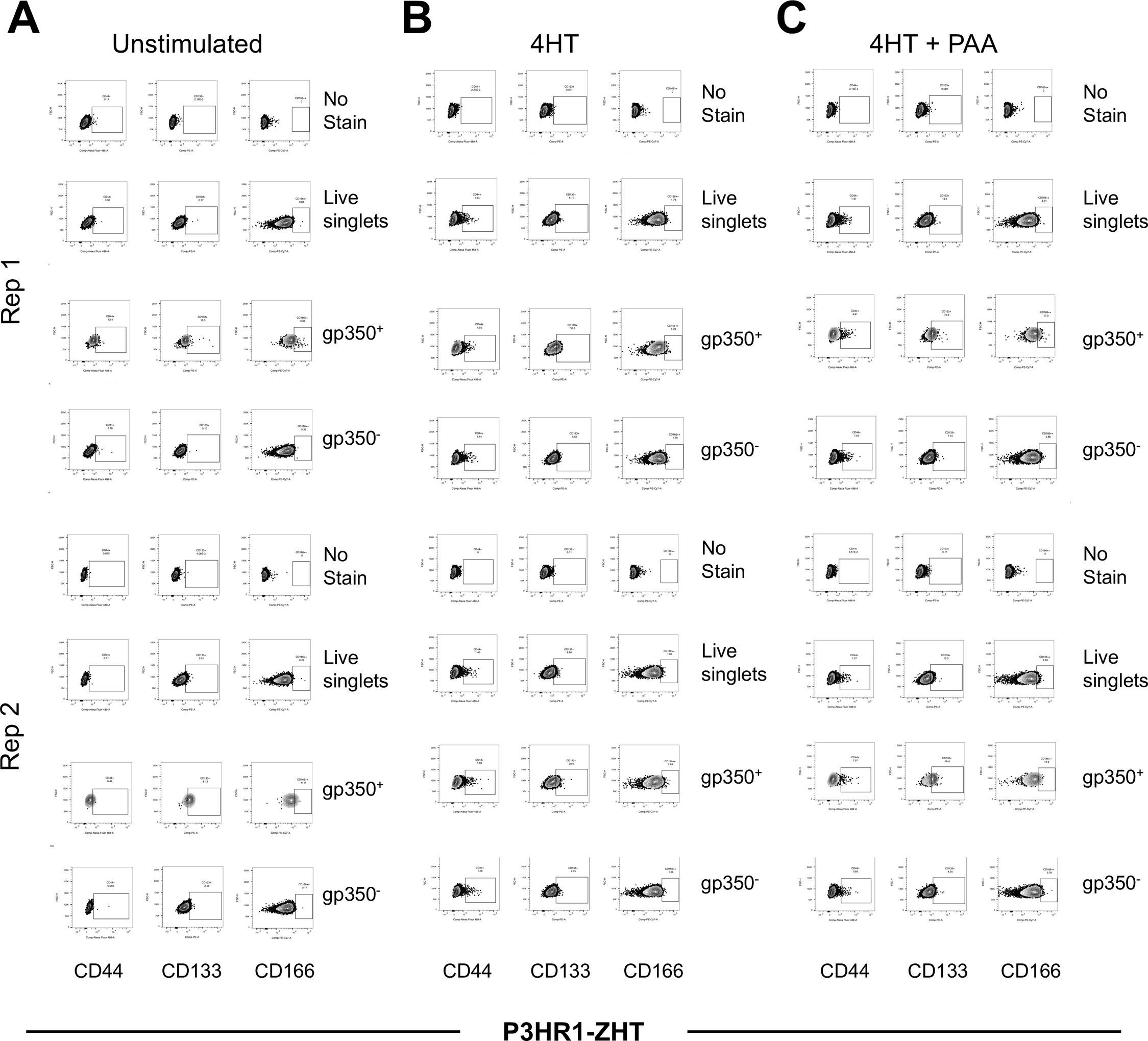

Supplement: S11 Fig — (A) Controls, gating, and stemness biomarker expression by gp350 status in unstimulated cells. (B) Controls, gating, and stemness biomarker expression by gp350 status in 4HT-treated cells. (C) Controls, gating, and stemness biomarker expression by gp350 status in cells co-treated with 4HT and PAA. (TIF) [file ppat.1012341.s011.tif]

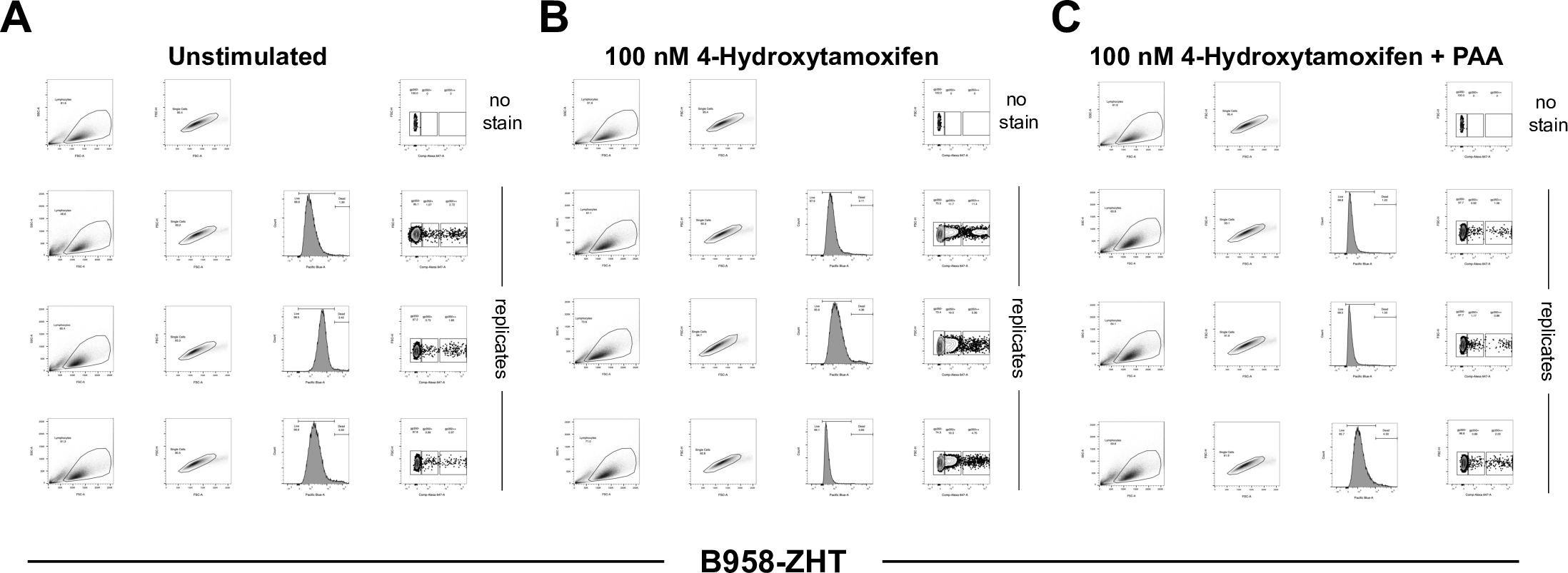

Supplement: S12 Fig — (A) Controls, gating, and gp350 expression in unstimulated cells. (B) Controls, gating, and gp350 expression in 4HT-treated cells. (C) Controls, gating, and gp350 expression in cells co-treated with 4HT and PAA. (TIF) [file ppat.1012341.s012.tif]

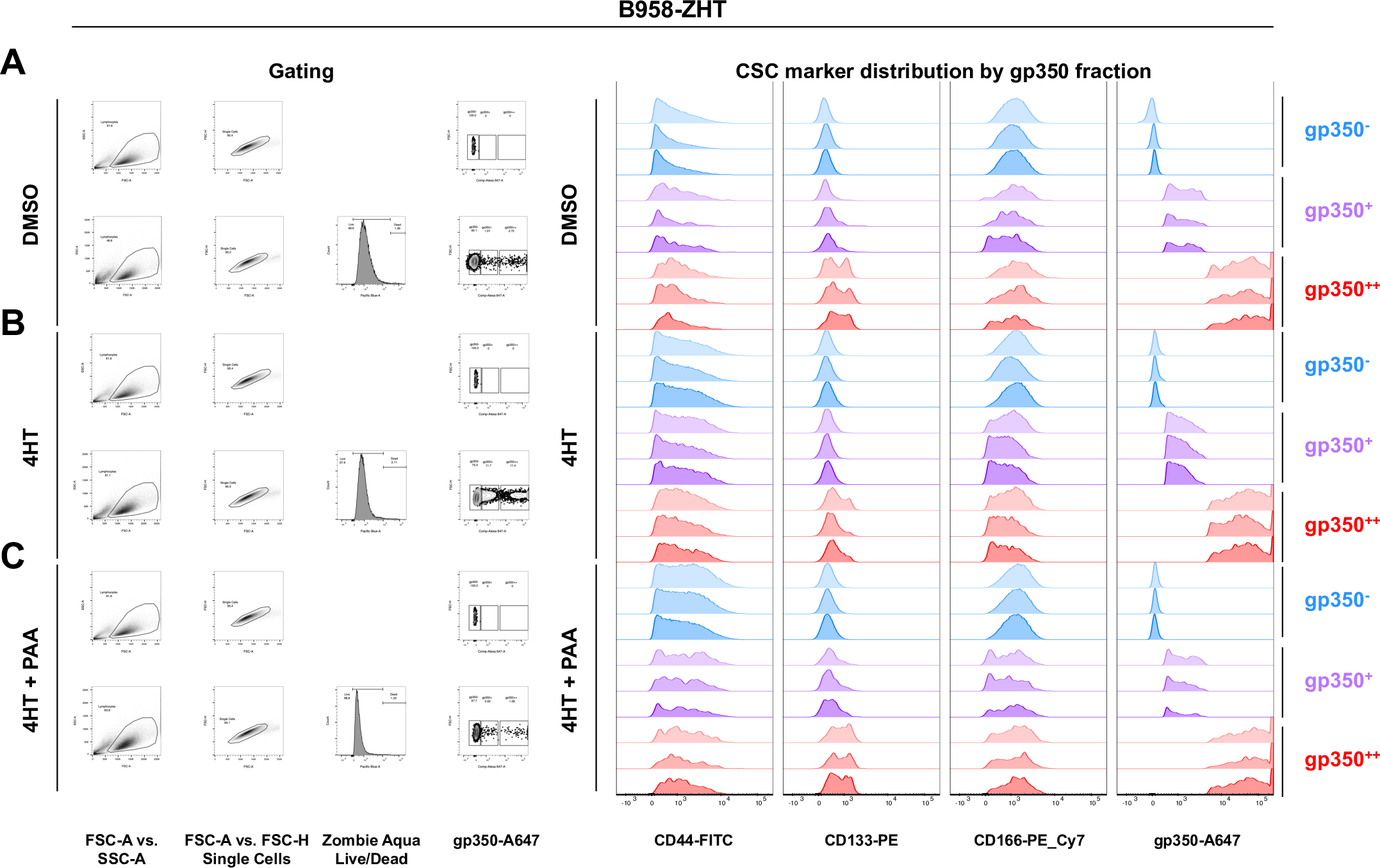

Supplement: S13 Fig — (A) Controls, gating, and stemness biomarker expression by gp350 status in unstimulated cells. (B) Controls, gating, and stemness biomarker expression by gp350 status in 4HT-treated cells. (C) Controls, gating, and stemness biomarker expression by gp350 status in cells co-treated with 4HT and PAA. (TIF) [file ppat.1012341.s013.tif]

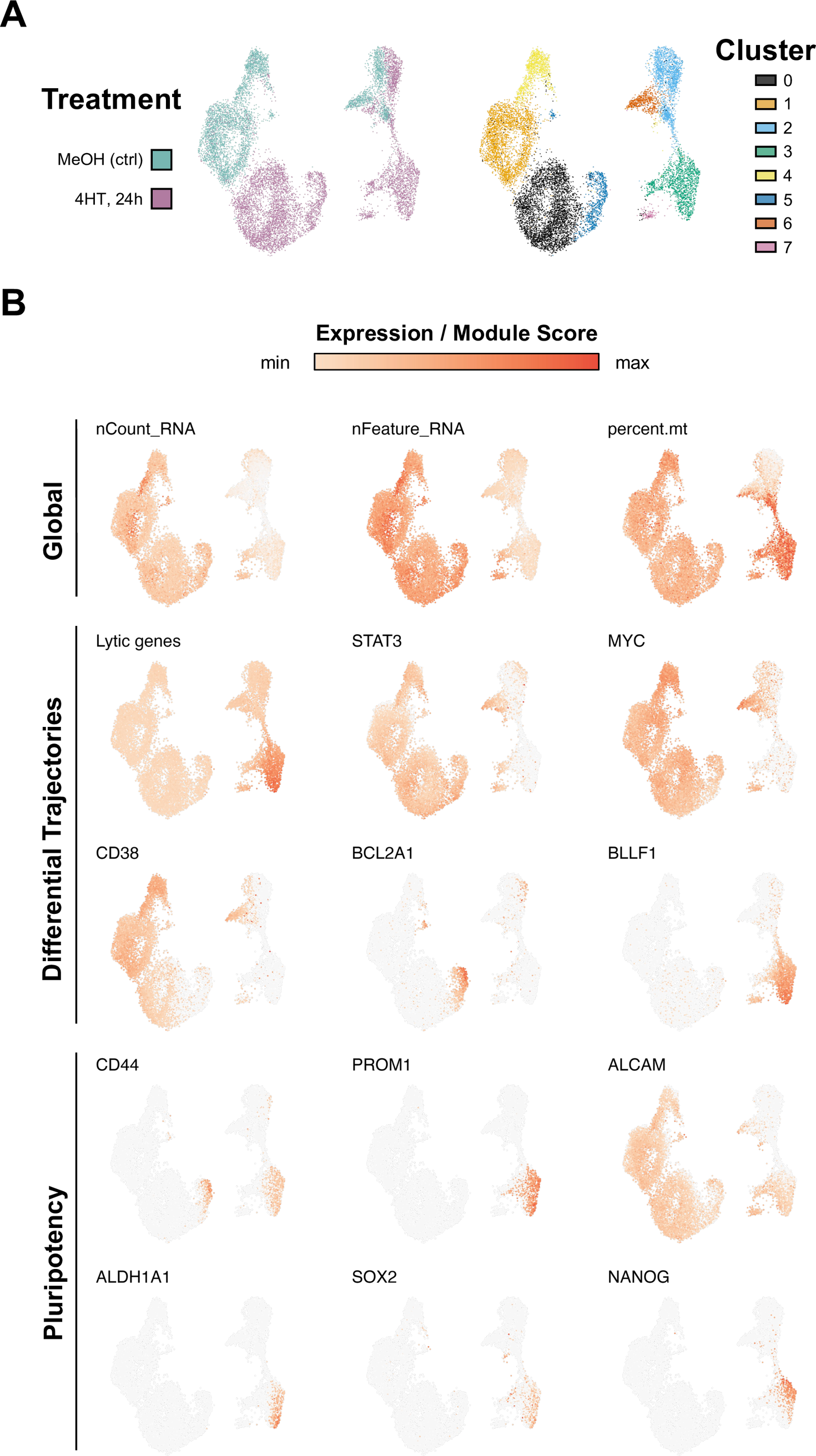

Supplement: S14 Fig — (A) Overview of P3HR1-ZHT replicate experiment treatments (methanol control and 4HT) and identified clusters. (B) UMAP visualization of global QC metrics (top row), differential abortive and lytic responses correlated with STAT3 and MYC levels (2nd and 3rd rows), and upregulated pluripotency signature in lytic cell subsets (4th and 5th rows). (TIF) [file ppat.1012341.s014.tif]
