# Supplementary material for: Epstein-Barr virus reactivation induces divergent abortive, reprogrammed, and host shutoff states by lytic progression
Source: PLoS Pathog. 2024 Oct 24;20(10):e1012341. doi: 10.1371/journal.ppat.1012341 (PMC11563402; doi:10.1371/journal.ppat.1012341)
Supplement: Attachment — Submitted filename: scLytic_PLoS_Path_author_responses_20240909.docx [file ppat.1012341.s020.docx]

**Title**: Epstein-Barr virus reactivation induces divergent abortive, reprogrammed, and host shutoff states by lytic progression

**ID**: PPATHOGENS-D-24-01229 - [EMID:da782b32cfe8054f]

**Author Responses to Reviewer Comments**

Original reviews in black

Author responses in blue

Three referees have reviewed the manuscript, each of whom appreciated strengths of the study, but each of whom have specific, focused points that will be important to address in a revised submission. In particular, reviewer #3 suggests additional supporting evidence from an additional model system (separate from the conditional P3HR-1 model).

We appreciate the thorough and constructive comments provided by each of the reviewers. Please see detailed responses below, which address these points. We hope the reviewers and editors share our view that the implementation of these recommendations and additional data have substantially strengthened the revised manuscript.

**Reviewer's Responses to Questions**

**Part I - Summary**
Please use this section to discuss strengths/weaknesses of study, novelty/significance, general execution and scholarship.

Reviewer #1: This manuscript from the Luftig lab uses single cell analysis to profile changes in B cells that occur upon reactivation of EBV from latent to lytic infection. This is an important question since EBV-positive B cells do not efficiently reactivate, but the reasons for this have not been clear. Using single cell analysis, they have been able to determine correlations between full reactivation and expression of various cellular proteins, suggesting which cellular proteins suppress reactivation. The results support previous studies that Myc suppress reactivation and that full EBV reactivation results in cell cycle arrest in early S. It also provides additional information, including upregulation of NF-kB and IRF3 occurring in abortive lytic cells and correlations of EBV protein expression with host shut-off. Finally, this study identifies stages of reactivation based on expression of EBV genes, showing that some cells enter the lytic cycle partly but not fully, referred to as abortive lytic infection. This is an interesting concept since previous dogma suggested that expression of EBV immediate early genes would be sufficient to activate full lytic infection. I only have minor comments for improvement.

We thank the reviewer for positive consideration of the manuscript. Minor comments are addressed below in Part III.

Reviewer #2: This study attempts to address a very interesting mystery in gammaherpesvirus biology, the impact of the host cell environment on the fate of productive viral replication. The authors performed single cell sequencing of two EBV+ B cell lymphoma cell lines, both during latency and following lytic induction. Cells at different stages of lytic replication were identified and pseudotime analysis was performed to interpret the dynamics of host cell gene expression at and between different stages of lytic progression. The authors identify subpopulations of fully lytic cells with a cancer stem cell gene expression program, as well as cells that are a. refractory to lytic entry and b. abortive following lytic entry. Each subpopulation is studied to better understand the underlying host gene expression programs that influence the fate of induced cells. In addition to the important conclusions drawn from this analysis, the data has the potential to serve as a valuable resource for researchers in the field.

The key conclusions of this work, however, hinge on accurate identification of refractory and abortive cells. Complementary approaches to validate subpopulations will provide more convincing evidence of their existence and lead to a more confident interpretation of their underlying characteristics.

We thank the reviewer for positive consideration of the manuscript. Please see specific responses in Parts II and III that describe additional experiments and analyses undertaken to address this concern and further support key conclusions.

Reviewer #3: The manuscript uses scRNA-seq methods to analyze the viral and host gene and protein expression patterns upon reactivation of EBV latency. The study focuses primarily on one highly engineered model P3HR1-ZHT where the BZLF1 immediate early transcriptional activator is constitutively expressed and under the regulation of 4-hydroxytamoxifen.
They identify 5 different response clusters. They find that abortive lytic cells have high NF-kB activity, lytic cells with stem-like pluripotency features, and genes that escape the transcriptional host-shut off during lytic reactivation. Finally, they provide corroborating data using a second model utilizing the B958-ZHT.

We thank the reviewer for their helpful comments, which are addressed in detail in Parts II and III.

**Part II – Major Issues: Key Experiments Required for Acceptance**
Please use this section to detail the key new experiments or modifications of existing experiments that should be absolutely required to validate study conclusions.

Generally, there should be no more than 3 such required experiments or major modifications for a "Major Revision" recommendation. If more than 3 experiments are necessary to validate the study conclusions, then you are encouraged to recommend "Reject".

Reviewer #1: (No Response)

Reviewer #2: - Cells are called refractory if they don’t express lytic genes following induction with 4HT. The authors detect high MYC expression in cells considered refractory, confirming a previous study (Guo et. al., 2020) that MYC expression prevents lytic reactivation. At the same time, MYC expression is reduced by host shut off after lytic commitment, which would also explain finding relatively low MYC levels in reactivating cells. Is the stochasticity of the system an explanation for the population that remain latent? Or is there a population of cells that are inherently refractive to reactivation, and cannot efficiently be induced?

**A.** This question of what makes a cell refractory is an interesting one. As shown in the figures below (for review purposes), the scRNA-seq data suggest that refractory vs. abortive and lytic responses are associated with the mitotic state of a cell during lytic induction. Specifically, cells that are actively cycling appear more likely to exhibit refractory responses (**Review Figure 1**). Comparing refractory cells (Cluster A cells in the HT-treated samples) with unstimulated Cluster A cells, we found mild enrichment of mitochondrial, ribosomal, and protein folding terms (**Review Figure 2**). These changes are more subtle than those exhibited in the abortive (yellow) and lytic (red) clusters, but do indicate shifts from the basal cell line states. It is possible that these changes reflect the gradual enrichment of G2M and S-phase cells (with elevated metabolic activity) within cluster A after treatment, since cells from the G1 population are more likely to transition to lytic reactivation.

**B.** To further address the question of whether a population of cells is inherently refractory, we stimulated P3HR1-ZHT cells with 4HT for 48 hours and stained for the presence of gp350 and ICAM1 (**see Main Fig 3F**). We sorted out the population of cells that did not express either gp350 (lytic) or ICAM1 (abortive) to achieve a pure population of refractory cells. We then re-stimulated these double negative cells and assessed the level of gp350 and ICAM1 expression 24- and 48- hours later. We observed that there was a population of cells that responded to stimulation from within this original refractory pool (**Review Figure 3**). Interestingly, we also observed that a similar percentage of these double negative, refractory cells expressed ICAM1 when left untreated for 24h compared to re-stimulation of this same population. These data support both that the cell cycle state impacts reactivation (eg those in S/G2/M proceeding to the next G1 become susceptible to reactivation) and that a population of refractory cells will become abortive following re-stimulation.

**C.** We wish to make one clarification to the reviewer’s characterization that “*refractory cells don’t express lytic genes following induction*,” since the data are not quite so absolute (NB: we do not make this claim in the manuscript). A limited set of viral lytic genes (13) were slightly enriched in refractory vs. unstimulated cells of cluster A (**Review Figure 4**). This indicates that refractory cells express some viral genes, but not to the extent that key signatures of abortive or lytic cells are elicited. This suggests that the refractory response is not simply a result of stochastic induction within the P3HR1-ZHT model. Collectively, these data support a role for a cell’s proliferative state at the time it receives a lytic stimulus in the resulting fate trajectory. (Speculative note: from the perspective of viral selective adaptation, it is perhaps biologically “rational” that a host cell already supporting viral genome replication through active cell division might not be so prone to go lytic as one that is resting).

**Review Figure 1: Cell Cycle Phase and Global Cluster Annotation by Reactivation Timepoints.** The top row shows cell cycle scoring for untreated, 24h, 48h, and 72h post-HT (left to right). G1 phase (blue), G2M phase (magenta), and S phase (yellow) assignments are presented. The bottom row shows global cluster designations for the same timepoints. Note that, after HT treatment, refractory cells that remain in cluster A (blue) predominantly exhibit G2M and S phase signatures. Also note that the abortive cluster, cluster C, increases over treatment time and the population stays stable compared to cluster A and E.

**Review Figure 2: GO enrichment from DE comparison of post-HT Cluster A vs. unstimulated Cluster A cells.** Terms correspond to expression signatures upregulated in refractory cells versus the unstimulated P3HR1-ZHT cell line. Note that the majority of terms are related to mitochondrial, ribosomal, and protein control activities. These may reflect enrichment of actively cycling cells (i.e., generic metabolic activities supporting proliferation) in cells that exhibit refractory responses.

**Review Figure 3: A percentage of cells from a pure refractory population will reactivate upon stimulus. A**. P3HR1-ZHT cells were stimulated with 4HT for 48 h and stained for gp350 and ICAM1. Pre-sort staining of these cells highlights the different fate trajectories: refractory, abortive and lytic. Cells defined as refractory (gp350-, ICAM1-) were sorted out and returned to culture. Flow plots show that refractory cells were negative for both surface markers. **B**. Cells from **A** were either plated in DMSO (top) or re-stimulated with 4HT (bottom) and assessed for gp350 and ICAM1 status 24h (and 48h, shown in **C**) later. From this refractory (double negative) population, both DMSO and 4HT re-stimulated cells expressed ICAM1. A percentage of originally refractory cells also progressed through the lytic cycle and expressed gp350. **C.** Bar graphs depicting ICAM1 expression across populations of cells, treatments, and timepoints.

**Review Figure 4: Refractory cells upregulate limited lytic expression versus unstimulated cells.** Differential expression analysis of refractory versus unstimulated cells in Cluster A identified a small set of EBV lytic genes upregulated after treatment. This demonstrates that the refractory response is not purely a result of stochastic go/no-go lytic activation in the P3HR1-ZHT model. Expression of BKRF4 by timepoint is provided as a representative example (red circle highlights refractory cells with upregulated expression at 24h). The full DE gene list for the comparison includes 13 EBV lytic genes and is provided as a supplementary .csv file.

- Cluster C was labeled as “abortive” based on early lytic gene expression. It is unclear if these cells were in a final abortive state or if at the time of collection, they were in an early reactivation stage and would have continued their progression to viral replication. An experiment that follows the trajectory of cells deemed abortive would be helpful to validate the identification of this population.

We thank the reviewer for this important consideration. **Figure 2B** in the original submission shows that the proportion of cells in Cluster A (blue) is relatively stable across 24, 48, and 72 h post-HT. While the frequency of abortive cells gradually accumulates over time (Cluster C, yellow), the frequency of lytic cells (red) notably *decreases* over the time course. These kinetics suggest that the cells that remain in the refractory state (or abortive state) cannot be simply attributed to a delay in the transition to full lytic reactivation.

To further explore this, we conducted new experiments to sort and re-culture HT-induced cells by gp350/ICAM1 status. We found that the refractory cells (cluster A) harbor a sub-population destined for the abortive cluster (cluster C) as evidenced by the emergence of ICAM1+/gp350- cells 24h after sorting of 4HT treated P3HR1-ZHT cells (48h treat/sort of ICAM1-/gp350-) (**Review Figure 3 above**). However, we were unable to consistently sort enough abortive cells (ICAM1+/gp350-) to replate and induce to visualize the fate of these cells.

- The authors find that a number of cancer related genes are induced in a cluster of cells with late lytic gene expression (Figure 4) and suggest the potential for fully lytic cells to be reprogrammed. They confirm expression using RNA Flow-FISH. However, during reactivation, others have reported substantial cellular transcription alterations, many of which are induced unproductive, non-coding transcripts which overlap annotated genes. It would be important to 1. demonstrate protein expression of SOX2 and/or ALDHA1 in late lytic subpopulations, and 2. show evidence that these subpopulations survive lytic replication.

We thank the reviewer for this very relevant and helpful suggestion. Based on the polyA capture chemistry used by the 10x Genomics scRNA-seq kit, we reasoned that successful capture and amplification of sequences with extensive readthrough might be inefficient due to internal positioning of the polyA sequence. We have attempted to obtain protein-level evidence for reprogramming genes detected by scRNA-seq and RNA Flow-FISH. We observed that P3HR1-ZHT cells treated with 4HT for 24 h lead to a detectable level of SOX2 protein expression via intracellular flow cytometry (**Review Figure 5** and **Main Supp Fig 10**). We simultaneously stained these cells for the early lytic protein BMRF1 to determine whether this protein expression was exclusive to lytic cells. We observed that a majority of SOX2+ cells were also positive for BMRF1, supporting our findings at the RNA level. We would also like to point back to our original **Figure 4F**, where we detected the CSC like markers CD133 and CD44 by protein flow in reactivated cells. Expression of these markers was higher in gp350+ cells compared to gp350- cells.

With respect to the survival of productively lytic cells, we wish to make two notes – one regarding literature and one related to results from the experiment mentioned above. First, a recent publication from our lab (Willard *et al*, PMID: 37971257) demonstrated that gp350^+^ cells sorted from novel spontaneously lytic BL lines/EBV strains remain viable in culture. Moreover, these cells re-establish the characteristic frequency of spontaneous reactivation of the parental cell line (the same is true from sorted gp350^-^ fractions). As a general matter (independent of reprogramming expression), those findings provide empirical support for the ability of lytic cells to survive.

­­­

**Review Figure 5: A small population of lytic cells express SOX2 at the protein level.** A. Flow plots of untreated and treated P3HR1-ZHT cells 24 h post stimulation. Flow plots show that a small percentage of induced cells express SOX2 and that most of these cells are lytic (BMRF1+). B. Bar graph depicting the percentage of cells that expressed SOX2 between treatment groups, in biological triplicate.

Reviewer #3: Overall
This is an impressive and impactful study that identifies new gene pathways, and confirms previously identified pathways implicated in the control of EBV lytic-latent balance. The application of single cell RNA-seq and Flow FISH are technically strong and appropriate for this type of study. The major limitation and concern is that the data relies primarily, if not exclusively, on one highly engineered model system using P3HR1 BL cell and viral strain, and the BZLF1 conditional ER fusion gene. How well does this system capture critical regulation of BZLF1 and other aspects of physiological cell response to various reactivation stimuli? This limitation should be addressed more directly and extensively in the Discussion section. The authors should consider providing additional experimental evidence that some of these major findings are also true in natural reactivation systems, such as anti-Ig stimulated Akata or other cell systems, particularly non-engineered LCLs or other EBV lymphoid tumor cell lines. Further, the B958-ZHT cell line is stated to be a marmoset line so it is difficult to understand how the genes are mapped to the human genome effectively.

We thank the reviewer for the positive consideration of the manuscript and constructive feedback. We have added the requested material on the limitations of the P3HR1-ZHT system to the discussion section. Pursuant to the reviewer’s request, the revised manuscript also includes data and analysis from an additional scRNA-seq experiment examining responses of the Akata cell line to anti-Ig stimulation. Data from this experiment are presented in **Figure 7** of the revised manuscript, which is also embedded below.

As shown in **Figure 7**, Akata cells stimulated with anti-Ig recapitulate key expression signatures corresponding to abortive and lytic cells, respectively. Overall, the induction efficiency in this system is much lower (as expected) for lytic and abortive responses. Notwithstanding, abortive and lytic phenotypes were successfully mapped from P3HR1-ZHT data to cells in the integrated Akata dataset. Expression signatures in abortive and lytic cells were broadly consistent with those identified in the P3HR1-ZHT and B958-ZHT models. For example, abortive cells co-expressing *EBI3*, *BCL2A1*, *CD83*, *CD40*, and other NF-κB signature genes were identified. Likewise, lytic cells co-expressing extensive reprogramming / plasticity (*ALDH1A1*, *PAX6*, *NEUROD1*, *ITGA6*, *EPCAM*, *GATA3*, *ALPP*, *LGR4*) and host shutoff (*LHX1*, *IL1R1*, *JAG1*) signatures were observed.

We have also included detailed analysis of the unstimulated P3HR1-ZHT sample, which exhibits a low frequency of spontaneously lytic cells (in the absence of HT). These cells are closely related to small subset with hallmarks of developmental pluripotency (see embedded **Review Figure 4 above**). While it is a minor point given the Akata results described above, we believe this also provides support for lytic-associated reprogramming in the absence of expression driven by the engineered construct in this cell model.

With respect to mapping B958-ZHT sequences against hg38, we take the reviewer’s point, but note the high sequence homology between the two species. Following up on this, we examined five genes selected from the reprogramming signature (*ALDH1A1*, *HES1*, *LHX1*, *PAX6*, and *SOX2*) using BLAST2 alignment between *Homo sapiens* and the common marmoset (*Callithrix jacchus*) (**Review Figure 6**). Percent identity across these genes (including intronic regions) was ~92%. This rate is likely higher within conserved exons. Given that reads captured from polyA transcripts yield 50 bp reads, it is reasonable that many captured transcripts will map efficiently to the human reference. Specifically, the average expectation of 4 mismatches (divergent positions; based on 92 per 100 base identity) between species means that ~25% of captures transcripts will have no more than 1 mismatch by Poisson statistics (see graph; lambda = 4; # simulations = 10,000); ~65% of transcripts would be expected to have 3 or fewer mismatches to the respective gene between species.

**Review Figure 6. Interspecies alignment of *LHX1* – *Homo sapiens* and *Callithrix jacchus***. Diagonal in the nucleotide-to-nucleotide dotplot demonstrates sequence match (top panel). Overall alignment of the *LHX1* locus yielded 91.75% identity across the 8,293 base query with 99% query coverage (bottom panel).

We have also included a representative interspecies alignment summary for *LHX1* for review below. Note the high nucleotide correspondence in the pairwise sequence dotplot. While the new results from the Akata anti-Ig stimulation experiment (see **Figure 7** below) more directly address the reviewer’s broader point about validation, we still wanted to address this point and believe the phenotype validation in B958-ZHT is justified.

**Figure 7 (Revised Manuscript). Validation of abortive and reprogramming gene expression signatures in Akata BL cells stimulated by anti-Ig.** (**A**) Integrated scRNA-seq data from Akata cells without stimulation (blue) and 24 h after anti-Ig treatment to induce lytic reactivation (rose). (**B**) Cluster and subcluster mapping from P3HR1-ZHT data to Akata data via transfer anchor integration. Clusters are colored as for P3HR1-ZHT and B958-ZHT datasets. Colors represent the reference phenotype with the maximal integration score for each cell. (**C**) Cluster-resolved expression in integrated Akata scRNA-seq dataset. (**D**) UMAP visualization of viral read fraction and representative markers of abortive (yellow) and reprogramming (red) signatures. *LHX1* and *IL1R1* are known escapees of host shutoff (**E**) GO enrichment of DE genes upregulated in anti-Ig-induced Akata lytic cells.

Specific Comments

Fig 6. Relating to the analysis of the B958-ZHT model as a corroborating system for EBV reactivation. First, this is a marmoset cell line, so that is a question of concern. Second, the activation of BZLF1 remains through the 5-HT system, which may be very different than natural physiological signals. Perhaps related to this, is that the UMAP for the B958-ZHT looks very different than that for the P3HR1-ZHT. While many of the gene correlations are conserved, the authors should also highlight the aspects that are different between these two cell and viral types, and why.

Please refer to the analysis and explanation above for B958-ZHT results and the new data from BCR stimulation as a more physiologic trigger for lytic reactivation in Akata.

Regarding differences in the UMAPs, internal relative structures (e.g., cluster shapes and inter-cluster distances) can be used to inform relatedness, however the global structure of the latent embedding is more arbitrary. Given that projections for P3HR1-ZHT and B958-ZHT were generated independently, differences in global structure should be expected. The key aspect is that genome-wide phenotypes from one model transfer with high scores to the other model (transfer anchor integration in **Figure 6**, and now in **Figure 7** for Akata).

However, we take the reviewer’s broader point that there are also differences between the P3HR1 and B958 lines, and that perfect correspondence should not be expected. For example, one (P3HR1) is a BL-derived line with constitutive MYC expression driven by somatic mutation and lacks EBNA2 expression, whereas B958 was transformed *in vitro* and is infected with EBV expressing functional EBNA2. These differences will naturally lead to variations in the exact phenotypes (and their proportions) present in each model. Pursuant to the reviewer’s recommendation, we have added a point addressing these differences to the discussion.

**Part III – Minor Issues: Editorial and Data Presentation Modifications**
Please use this section for editorial suggestions as well as relatively minor modifications of existing data that would enhance clarity.

Reviewer #1: 1. The authors conclude that clusters C and D are incomplete reactivation states, as compared to cluster E, which is complete lytic infection, and refer to this as abortive lytic infection. However, I am unclear as to exactly which EBV genes are turned on in these clusters, and whether clusters C and D only express some immediate early and early genes without late gene, as suggested by the name abortive lytic. It appears that there is some BLLF1 (gp350) detected in C and D, indicating some late protein expression. Figure 2D is a very nice presentation of some of the changes occurring in the different clusters but only shows 5 EBV genes, whereas I assume most EBV genes would have been detected in cluster E. To better understand the different stages/subtypes of EBV lytic infection, it would be informative to include a representation similar to Fig 2D that shows all the EBV transcripts detected.

We have included the requested dot plot (viral gene expression by cluster) as a supplementary figure [**FIGURE S6**] in the revised manuscript.

2. The authors discuss their single cell profiles in relation to host shutoff and genes that escape host shutoff. However, it is not clear to me what they are using as a measure of host shutoff itself. Is this total cellular mRNA levels? This needs more explanation. Also they state that BGLF5 expression correlates with host shutoff, in keeping with a previously reported role of this protein in shutoff. However there is evidence that other EBV proteins might also play a role in host shutoff. Was BGLF5 the only EBV transcript that correlated with host shutoff in their data? If not a more complete analysis of the relationship of EBV proteins to host shutoff should be presented.

Thanks to the reviewer for pointing this out. We indeed use the reduction in cellular mRNA levels (mRNA captured per cell) as one global indicator of host shutoff (evident in **Figure 2C** and **Figure 4A**). *BGLF5* expression correlates with this shutoff as the reviewer notes, and the biological functions of the BGLF5 protein have the most direct relation to the reduction of mRNA levels we observe. As can be seen in the new dot plot of all viral genes [**FIGURE S6**], we also observe an increase in expression of *BSLF2/BMLF1* (EBV SM), which mediates a reduction in functional cellular gene product expression by exporting non-spliced host mRNA. However, we note that scRNA-seq is not appropriately suited to evaluate host shutoff associated with this biochemical function, as unspliced mRNA retaining polyA sequences may still be captured and sequenced.

As a note, we previously addressed mechanisms of nuclear chromatin reorganization underlying major changes in expression in the Discussion section (references 69 and 70 are particularly relevant). We have updated this discussion to directly reference a recent review by Casco & Johannsen, which nicely summarizes evidence for lytic-induced chromatin compaction as a non-canonical (BGLF5-independent) host shutoff mechanism [Casco, 2023 #170].

Reviewer #2: - The authors use PAA to model an abortive lytic cellular environment. Although PAA treatment blocks lytic progression, it is unlikely to mimic the underlying cellular environment that promotes abortive lytic replication.

Thank you to the reviewer for this important point. We have noted this distinction in the Discussion section of the revised manuscript.

- It would have helped to have had both the raw sequencing data and a gene expression matrix from the single cell sequencing experiments

As of this response, we have initiated a NIH GEO submission for FASTQ and UMI count matrices from all single cell experiments. These data will be publicly available shortly.

Reviewer #3: Lines 103-119. References may need to be re-aligned with statements.

Thank you to the reviewer – we have re-examined the references in these lines, which relate to key aspects of *BZLF1* and *BRLF1* function, activation, and contributions to disrupted host expression. After this review, we believe each reference is cited appropriately. If the reviewer has specific suggested changes, we are open to considering them.
